# Mapping the landscape of immunonutrition and cancer research: a correspondence on bibliometrics analysis

**DOI:** 10.1097/JS9.0000000000001000

**Published:** 2023-12-14

**Authors:** Jiawen Wang, Xinhao Wang, Yaoguang Zhang, Jianye Wang

**Affiliations:** a Department of Urology, Beijing Hospital, National Center of Gerontology, Institute of Geriatric Medicine; b Graduate School of Peking Union Medical College, Chinese Academy of Medical Sciences, Beijing, People’s Republic of China

*Dear Editor,*

In 1992, Daly *et al*.^1^ applied an immunonutrition (IMN) formula containing arginine, ω-3 polyunsaturated fatty acids, and nucleotides to postoperative patients with upper gastrointestinal tumors, and found that patients receiving IMN therapy had fewer injuries and inflammatory complications. Since then, research on IMN therapy for tumor patients has continued to emerge, forming an important branch of oncology nutrition – tumor immunonutrition – and showing broad application prospects^2^. Nowadays, nutritional support for malignant tumors is no longer an adjuvant therapy but a first-line treatment capable of improving patient prognosis and prolonging life expectancy^3^.

Recently, *International Journal of Surgery* published an article titled ‘Mapping the landscape of immunonutrition and cancer research: a comprehensive bibliometric analysis on behalf of NutriOnc Research Group’^4^. With great interest, we read the results of this study. We greatly appreciate the authors’ work in this field. However, we have some questions about the retrieval procedure that we would like to discuss with the authors.

First, this article is ambiguous in its description of the search strategy. The keywords used in the search strategy mentioned in this study are ‘immunonutrition’ and ‘cancer’. Common IMNs such as glutamate, arginine, ω-3 polyunsaturated fatty acids, and nucleotides were not mentioned. This may make some articles missing and lead to less accurate results in bibliometric analysis. In addition, the researcher was silent on the type of literature included. Generally, the type of literature included needs to be screened.

Second, the authors searched for documents published between 1 January 1998 and 15 May 2023 in IMN and cancer. This search strategy is suitable for situations where there are fewer early studies in the field and the time span is longer. However, in the case of this study, this may have resulted in the loss of some important seminal studies, such as^1^ and^5^. In the discussion, the authors mentioned that scientific research can be categorized into four stages, that is, the initial stage, the great development stage, the mature stage, and the completion stage. Obviously, the initial stage of the field in this study is incomplete.

Third, there is a lack of assessment of the quality of the literature. Missing keywords, misspellings, abbreviations, synonyms, etc. may affect the results of keyword co-occurrence analysis and hotspot analysis, and data cleaning is needed to get accurate results. This is a missing component in many bibliometric studies^6^. In fact, data quality control is of utmost importance for any research. We hope to draw the attention of similar articles to quality control in bibliometrics.

Although the above-mentioned concerns exist, this article may be the first to use bibliometric analysis to review and guide future IMN and cancer research. We thank the authors for their work in the field related to IMN and cancer.

## Ethical approval

Not applicable.

## Consent

Not applicable.

## Sources of funding

Not applicable.

## Author contribution

J.W. and X.W.: writing; Y.Z. and J.W.: study design.

## Conflicts of interest disclosure

There are no conflicts of interest.

## Research registration unique identifying number (UIN)

Not applicable.

## Guarantor

Jiawen Wang.

## Data availability statement

Not applicable.

## Provenance and peer review

Not commissioned, externally peer-reviewed.
